# Orthodontic Management of Class II Malocclusion with Clear Aligners: Mandibular Advancement vs. Class II Elastics

**DOI:** 10.3390/children12050562

**Published:** 2025-04-26

**Authors:** Elisabetta Cretella Lombardo, Letizia Lugli, Roberta Lione, Patrizio Bollero, Paola Cozza, Chiara Pavoni

**Affiliations:** 1 Department of Health Science, UniCamillus-Saint Camillus International Medical University, 00131 Rome, Italyroberta.lione@unicamillus.org (R.L.); paola.cozza@unicamillus.org (P.C.); 2 Department of Systems Medicine, University of Rome “Tor Vergata”, 00133 Rome, Italy

**Keywords:** Class II, aligners therapy, cephalometric analysis

## Abstract

**Background**: This cephalometric study aimed to evaluate the effects of clear aligner therapy in growing individuals with Class II malocclusion, comparing two functional approaches: the use of Class II elastics or the Mandibular Advancement (MA). **Methods**: Cephalometric data from 39 patients with Class II malocclusion treated using clear aligners either combined with Class II elastics (EL group; n = 18) or Mandibular Advancement (MA group; n = 21) were analyzed and compared with an untreated control group (UC2; n = 15). **Results**: Both treatment groups (EL and MA) showed a significant reduction in the ANB angle compared to the control (MA: −1.5°; EL: −2.2°; UC2: +0.2°). An increase in mandibular length, as measured by Co–Gn, was observed in both the EL and MA groups (+5.5 mm and +8.3 mm, respectively) relative to the control group. Soft tissue analysis of the Pg–TVL distance from T1 to T2 revealed the most substantial forward displacement of the chin in the MA group (MA: +2.0 ± 3.7 mm; EL: +0.5 ± 0.7 mm; UC2: −1.6 ± 3.3 mm). Vertically, the MA group exhibited a more marked decrease in the palatal-mandibular plane angle than the other groups. Both treatment modalities significantly reduced overjet and overbite from T1 to T2. **Conclusions**: The EL and MA appliances effectively advanced the mandible, leading to significant improvements in the sagittal relationship, overjet, and overbite while maintaining stable vertical control. Additionally, the MA group exhibited a more pronounced forward movement of the soft tissue chin.

## 1. Introduction

Class II malocclusion may arise from an excessive forward position of the maxilla, either skeletal or dental in nature, a backward-positioned mandible, or a combination of these factors [1,2,3].

Various treatment strategies for Class II malocclusions have been explored in the literature, including distalization [4], extraoral traction, dental and skeletal expansion, functional orthopedic devices, fixed appliances, and intermaxillary elastics [5].

For Class II, Division 1 malocclusions characterized by skeletal mandibular retrusion, functional therapy is commonly used to stimulate mandibular growth. This approach advanced the mandible forward while simultaneously improving chin position [6].

Various functional devices have been developed to promote mandibular growth and facilitate its forward repositioning [7,8,9,10,11].

One of the most widely utilized functional appliances is the Twin Block (TB), developed by Clark. It consists of two removable plates with inclined acrylic bite blocks that interlock, encouraging the mandible to move forward during bite closure [12].

Among these, the Twin Block (TB), created by Clark, is one of the most widely used. This appliance is composed of two interlocking removable plates with inclined acrylic surfaces that guide the lower jaw into a forward position during bite closure [12].

Several studies have revealed that functional appliances can induce an elongation of the mandible, with the best results obtained at pubertal or immediately post-pubertal periods of skeletal development [6,7,8,10,11,12,13,14,15,16,17,18,19,20].

In 2017, Align Technology introduced the Invisalign® Mandibular Advancement (MA) appliance (Align Technology, based in San Jose, CA, USA), which was designed to integrate skeletal growth modification with simultaneous anterior dental movement. This innovation combined the previously distinct phases of functional orthopedic therapy and orthodontic alignment into a unified treatment protocol. The Invisalign^®^ Mandibular Advancement (MA) replicates the mechanism of action of functional appliances as it moves the lower jaw forward. It does so with inclined planes built into buccal precision wings placed between the first molar and premolars, which can only interlock when the patient pushes the mandible forward [20].

In recent years, a few case reports have suggested that intermaxillary elastics applied to clear aligners in growing patients can be effective in correcting Class II malocclusion, with similar effects to functional appliances [21,22].

The Invisalign® Mandibular Advancement (MA) appliance replicates the action of traditional functional devices by positioning the mandible forward. This effect was achieved through the incorporation of inclined planes within buccal precision wings, located between the first molars and premolars, which engaged exclusively when the patient advanced the lower jaw [20].

In recent years, several case reports have indicated that the use of intermaxillary elastics in conjunction with clear aligners in growing patients may effectively correct Class II malocclusions, producing results comparable to those of traditional functional appliances [21,22].

To our knowledge, only one study, published by Dianiskova et al. [23], has analyzed the effects of Class II elastics on aligners in comparison with fixed multibracket therapy, showing that both systems produced a similar correction of sagittal discrepancies in growing patients.

However, no studies have evaluated the effects of Class II elastics on clear aligners with respect to the MA.

Therefore, the purpose of the present retrospective cephalometric study was to analyze the dental, skeletal, and soft tissue changes induced by clear aligners for the resolution of Class II malocclusion in growing patients with Class II elastics or Mandibular Advancement (MA) when compared with an untreated Class II control group.

The null hypothesis tested was that both types of treatment were equally effective in inducing an orthopedic effect on Mandibular Advancement in growing patients.

## 2. Materials and Methods

Cephalometric records were retrospectively analyzed for 39 growing patients with Class II, Division 1 malocclusion who underwent consecutive treatment with either Class II elastics (EL group: n = 18; 8 males, 10 females; mean age 11.2 ± 1.1 years) or the Mandibular Advancement appliance (MA group: n = 21; 10 males, 11 females; mean age 11.1 ± 1.2 years).

The study received approval from the Ethics Committee of the Hospital of Rome Tor Vergata (Protocol No. 201/16), and informed consent was obtained from the parents of all participating subjects.

In this retrospective controlled clinical trial, sample size estimation was performed using an analysis of variance (ANOVA), aiming to detect a 1.7° difference in the primary outcome measure, the ANB angle. The calculation assumed a standard deviation of 1.4° [24], a significance level (α) of 0.05, and a statistical power of 80%.

As a result, a minimum of 15 participants per group was necessary (Sigma-Stat 4.0, Systat Software Inc., San José, CA, USA).

### 2.1. Inclusion and Exclusion Criteria

Participants for the study group were chosen based on specific inclusion criteria, including European ancestry (Caucasian), an overjet exceeding 5 mm, a bilateral full Class II or end-to-end molar relationship, an ANB angle greater than 4°, and a noticeable enhancement in facial profile when the mandible was positioned forward. Furthermore, participants were required to be at stage 3 of cervical vertebral maturation (CVM) at the start of treatment (T1) and have available lateral cephalograms taken at two time points: T1, marking the beginning of treatment, and T2, recorded at the end of therapy.

A total of fifteen untreated Caucasian subjects with Class II, Division 1 malocclusion were selected from the American Association of Orthodontists Foundation Craniofacial Growth Legacy Collection (http://www.aaoflegacycollection.org) (Accessed on 5 January 2023). These individuals constituted the UC2 group (n = 15; 4 males, 11 females; mean age: 10.9 ± 1.1 years).

Both the treated and control groups were selected according to their skeletal maturity, evaluated at T1 using the cervical vertebral maturation (CVM) method. This approach, which offers an alternative to hand–wrist radiographs, enables the assessment of skeletal development in growing individuals. CVM staging was performed by an experienced examiner [25].

The three groups were matched in terms of age and gender distribution. Demographic characteristics for the MA, EL, and UC2 groups are summarized in Table 1.

All patients received treatment from expert clinicians with comparable clinical expertise.

### 2.2. Treatment Protocol

Subjects in the MA group underwent treatment using the Mandibular Advancement (MA) feature, which incorporated precision wings within the aligners to progressively reposition the mandible forward. These wings facilitated an incremental shift, offering an advantage over traditional functional appliances by simultaneously enabling mandibular repositioning and tooth movement, thereby shortening treatment duration.

Constructed from the same material as the aligners, the precision wings included additional reinforcements, such as grooves and structural modifications, to reduce flexibility and enhance rigidity.

The ClinCheck protocol followed a structured three-phase approach: Pre-MA phase, MA phase, and Transitional phase.

- The **Pre-MA phase** was initiated automatically in cases of an overbite greater than 7 mm or molar rotations exceeding 20° and crossbite to optimize wing placement or facilitate the first stage of Mandibular Advancement. In this phase, the treatment plan focused on expanding the maxillary arch and rotating the upper first molars distally in reference to the Ricketts line. Simultaneously, in the lower arch, the strategy aimed to level the curve of Spee and retrocline the lower incisors to establish adequate overjet for the subsequent Mandibular Advancement.
- In the **MA phase**, the precision wings were designed to provide an incrementally forward shift of 2 mm for every eight aligners, ensuring controlled and progressive Mandibular Advancement. The final setup during MA phase was taken with the upper and lower incisors positioned edge-to-edge.
- After the Mandibular Advancement was completed, the **Transitional phase** was initiated to stabilize the mandible in its forward position during the interval before the delivery of standard or refinement aligners.

**Figure 1:**
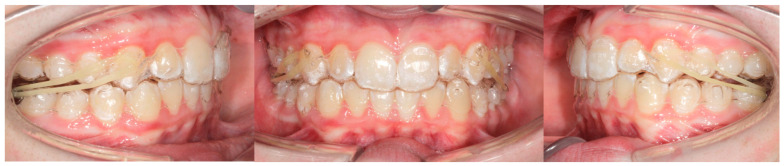
Frontal and lateral views of a Class II elastics appliance.

In the EL group (Figure 1), the ClinCheck programming included an initial expansion of maxillary and mandibular arches to recover the proper symmetric transverse dimension. The correction of the sagittal Class II relationship was performed by using intermaxillary Class II elastics to promote an anterior repositioning of the mandible. The final configuration with Class II elastics involved a molar and canine class I relationship.

Additional treatment goals involved leveling and aligning, improving the facial profile, and obtaining a natural lip position.

After expansion, to provide retention for interarch elastics use, precision cuts were designed on the aligner’s surface. Elastics were hooked directly from precision cuts on the upper canines to the cut on the lower first molars. 

Patients were instructed to wear 6 oz, ¼” Class II elastics full-time.

In both groups, interproximal reduction (IPR) was planned on the lower arch, promoting the control of the inclination of the lower incisors to recover the amount of overjet necessary for forward displacement of the mandibular.

Attachments were engineered by Align Technology™ to achieve predictable tooth movements and placed according to the Align Technology™ attachments’ protocol [26]

Patients were instructed to wear the aligners for 20–22 h per day and to change them once a week. They returned every six weeks for a check of the aligners’ fit and attachments’ position. Both treatments were discontinued after the achievement of a Class I molar relationship.

### 2.3. Cephalometric Analysis

For each patient, lateral cephalometric radiographs were obtained at two time points—T1 (before treatment) and T2 (after treatment)—using a cephalostat with a 1.5 m focus-to-film distance. To ensure consistency, all cephalograms were standardized by setting the magnification factor to 0%.

Cephalometric evaluation was performed using Viewbox (version 4.0, dHAL Software, Kifissia, Greece), with a customized digitization protocol designed specifically for analyzing the lateral radiographs.

**Figure 2:**
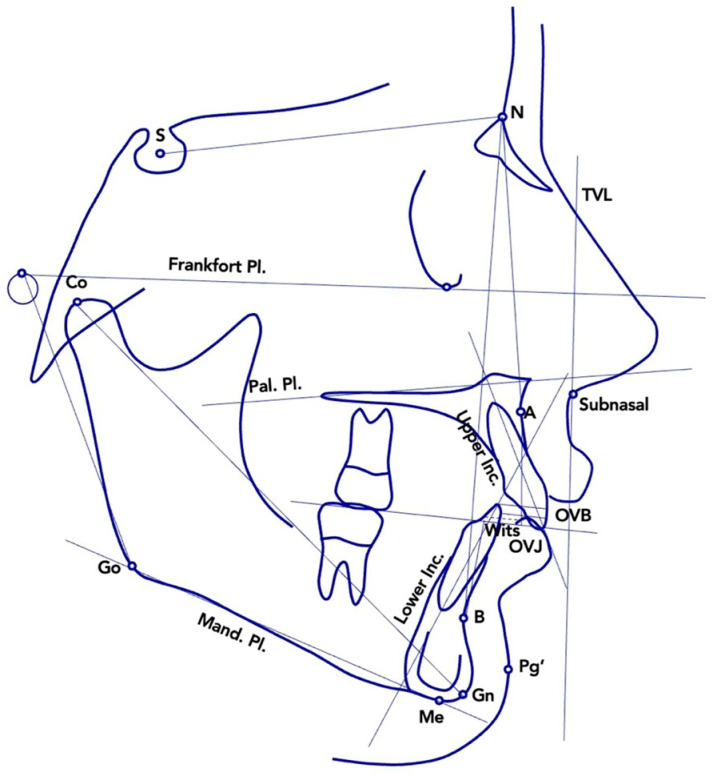
Cephalometric variables measured at T1 and at T2. Abbreviations: S—sella; N—nasion; Pal—palatal; Pl.—plane; Mand.—mandibular; Inc.—incisor; OVJ—overjet; OVB—overbite (vertical overlap); TVL—true vertical line (perpendicular to the Frankfort Plane, passing through subnasale); Go—gonion; Co—condylion; Me—menton; Gn—gnathion; Pg’—soft tissue pogonion; A—point A; B—point B.

At T1 and T2, cephalometric measurements were carried out to compare the three study groups (MA, EL, and UC2). A single investigator (LL) performed the digital tracing of the lateral cephalograms in one session. To ensure accuracy, another examiner (CP) reviewed the anatomical landmarks and outlines. Any discrepancies in landmark identification were resolved through mutual agreement. The cephalometric reference points, lines, and angles used in the study are illustrated in Figure 2.

### 2.4. Statistical Analysis

To assess differences in gender distribution among the three groups, Fisher’s exact test was applied. Descriptive statistics and comparative analyses for the EL, MA, and UC2 groups were carried out at T1 (baseline) and for the changes observed between T1 and T2 using analysis of variance (ANOVA) followed by Tukey’s post hoc test. When the data did not meet the normality distribution, as determined by the Shapiro–Wilk test, the Kruskal–Wallis test was employed, with Dunn’s post hoc analysis used for pairwise comparisons [27].

### 2.5. Method Error

To evaluate the accuracy of the method, all cephalometric measurements were conducted by a trained examiner (LL) with four years of experience. To verify reliability, 20 radiographs were reanalyzed after a two-week interval. Intra-observer consistency was measured using the intraclass correlation coefficient (ICC), while random error was assessed through the method of moments’ estimator (MME) [28].

## 3. Results

The intraclass correlation coefficients (ICCs) ranged from 0.720 to 0.993, reflecting a substantial to nearly perfect level of inter-rater reliability [28]. The method error (MME) for angular measurements ranged between 0.3° and 1.0°, while for linear measurements, it varied from 0.3 mm to 0.8 mm.

There were no statistically significant differences in gender distribution among the three groups, as indicated by Fisher’s exact test (*p* = 0.114). Demographics data for the groups are reported in Table 1.

The duration of the treatment was similar for both groups (EL group: 2.1 ± 0.3 years; MA groups: 2.2 ± 0.2 years; unpaired *t* test *p* = 0.223).

As reported in Table 2, the baseline assessment at T1 demonstrated statistically significant differences between the three groups exclusively in vertical overbite. No notable differences were detected in other linear or angular measurements at this stage.

Table 3 presents the descriptive statistics and statistical comparisons of the T2–T1 changes.

A statistically significant and clinically relevant reduction in the ANB angle was recorded in both the MA and EL groups when compared to the UC2 group (MA group: −1.5° ± 1.4°; EL group: −2.2° ± 1.7°; UC2 group: +0.2° ± 0.3°).

Regarding the Co-Gn measurement, both the MA and EL groups exhibited statistically significant increases compared to the UC2 group, with a total of +8.3 mm in patients treated with the MA appliance and of +5.5 mm in EL patients. In addition, MA patients showed a statistically significant increase in linear distance from the condylion to the gnathion of 2.8 mm in comparison with subjects treated by means of EL.

For soft tissue evaluation, the increase in Pg-TVL distance from T1 to T2 was notably different across the three groups, with MA demonstrating the most pronounced forward movement of the soft tissue pogonion (MA: +2 mm ± 3.7 mm; EL: +0.5 mm ± 0.7 mm; UC2: −1.6 mm ± 3.3 mm).

In the vertical dimension, the angle between the palatal and mandibular planes showed a more significant reduction in the MA group compared to the EL and UC2 groups. 

A significant reduction in overjet was observed between T1 and T2 in the MA and EL groups, in contrast to the control group, which showed no change (MA: −1.5 ± 7.0 mm; EL: −1.8 ± 2.5 mm; UC2: +0.0 ± 0.0 mm).

In the MA group, the overbite value significantly decreased compared to the EL group and UC2 group (MA group: −1± 7.9 mm; EL group: +0.9 ± 2.1 mm; UC2 group: +0.7 ± 1.6 mm).

## 4. Discussion

Recent advancements have expanded the scope of aligner systems, enabling the treatment of various types of malocclusions, including Class II cases. The recent literature suggests that Class II elastics used in conjunction with clear aligners in growing patients can achieve effects comparable to those of traditional functional appliances [22,23,29,30]. 

In the literature, several studies have analyzed the effectiveness of MA treatment [20,31,32,33,34,35,36,37,38,39]. However, to our knowledge, no studies have assessed the effects of the MA with respect to Class II elastics on clear aligners. 

Therefore, the aim of the present retrospective cephalometric study was to assess the effects of Mandibular Advancement treatment performed with the Invisalign Mandibular Advancement protocol compared to a standard protocol with intermaxillary elastics applied on clear aligners in growing patients. 

Our results indicate that both treatment modalities effectively corrected Class II malocclusions, as evidenced by improvements in the ANB angle in both the EL and MA groups compared to the untreated controls. A statistically significant difference among the groups was observed in chin advancement, assessed by measuring the distance of the soft tissue pogonion from the true vertical line (TVL). The findings of this study indicate that both EL and MA appliances effectively manage Class II malocclusion, with MA demonstrating a more pronounced enhancement of the facial profile.

The MA and EL groups induced an orthopedic effect with mandible forward repositioning and facial profile improvement, which was more significant in patients with Mandibular Advancement. However, the MA appliance seemed to be more efficient in the elongation of Co-Gn, in promoting chin advancement and, consequently, in enhancing the facial profile. This could be related to the protrusive position of the mandible achieved by using the MA compared to the virtual jump realized with the elastics. 

The effectiveness of the MA protocol may also be attributed to its full-time application, ensuring sustained Mandibular Advancement throughout the day. In contrast, the success of elastics depends significantly on patient cooperation.

With both techniques, there was a reduction in the overjet with good control of the incisor’s inclination. Several studies have demonstrated that the combination of clear aligners and Class II elastics can effectively control the positioning of lower incisors and overall dental alignment, providing significant improvements in Class II malocclusions [40,41,42].

In accordance with the results reported by Daniskova et al. [23] in 2020, the present retrospective study revealed that aligners with Class II elastics represent an important viable therapeutic option for specific patients where preservation of the lower incisor position is required. It is likely that the greater control occurs thanks to correct digital planning with proper IPR performed in the lower arch during treatment. This is associated with the resistance to deformation of the aligner, which keeps the entire arch locked, with a better distribution of forces exerted by the elastics on the aligner in comparison to their effect when used with brackets [40,41,42]. 

Moreover, as reported in the literature [43,44,45,46], the use of Class II elastics on aligners can provide an additional advantage by eliminating occlusal interferences during anteroposterior movement because of the occlusal thickness of the appliance.

This allows for greater freedom of mandibular movement, facilitating mandibular mesial repositioning during pubertal growth spurts. Moreover, interarch biomechanics with Class II elastics could help to restore the correct sagittal relationship through functional rebalancing of the oral and perioral muscles.

Indeed, as assessed by Ra’ed Al-Dboushin in 2024, clear aligner therapy can reduce occlusal interferences and help manage muscular activity, providing a stable occlusal environment for anteroposterior movements of the arches [45].

In the vertical plane, there was a significant reduction in overbite in the MA group compared to the other two groups at T2. However, it is important to emphasize that at T1, the MA group had a deep overbite.

An additional potential advantage of clear aligner therapy is the bite-block effect, which may enhance vertical control, as supported by findings from previous studies [45,46,47,48,49].

As widely reported in the literature, Class II elastics used in conjunction with fixed appliances typically induce molar extrusion and contribute to a clockwise rotation of the occlusal plane [50,51,52,53]. This effect of intermaxillary elastics was first reported in 1997 by Tulloch et al. [53] in a randomized clinical trial. This study analyzed the effects of early orthodontic intervention, including the use of Class II elastics, on skeletal morphology in Class II patients. The authors found that the use of elastics influences not only the vertical position of molars but also mandibular orientation, inducing mandibular post-rotation.

For this reason, clear aligner therapy could represent a suitable treatment strategy in patients with an increased vertical dimension.

One limitation of this cephalometric study is its short-term follow-up and the relatively small sample size. Nevertheless, given the recent introduction of these new techniques, additional research is warranted to expand the cohort and assess the long-term stability of the outcomes.

## 5. Conclusions

The comparison between EL, MA, and UC2 for the treatment of Class II malocclusion in growing patients produced, in the short-term period, the following conclusions:

- Both the MA and EL protocols are effective in the correction of Class II malocclusion and in the reduction in the overjet value, evaluated cephalometrically, when compared with untreated control group (MA group: −1.5 ± 7.0 mm; EL group: −1.8 ± 2.5 mm; UC2 group: 0.0 ± 0.0 mm).
- MA treatment induced a significantly greater advancement of the chin associated with an improvement in the facial profile when compared with the EL and UC2 groups (MA group: +2 mm ± 3.7 mm; EL group: 0.5 mm ± 0.7 mm UC2 group: −1.6 mm ± 3.3 mm).
- Both appliances seemed to ensure good control of the dental inclination of the incisors, allowing the mandible to be advanced.

However, the null hypothesis was rejected: both treatments were effective in inducing significant Mandibular Advancement compared to the control group; however, the orthopedic effect was shown to be greater in the MA group compared to the EL group. The reason for this could be the time of application of Class II elastics, which is collaboration-dependent.

## Figures and Tables

**Table 1:** Demographics for the treatment and control groups.

	T1 Mean Age ± SD	T2 Mean Age ± SD
MA Group (n = 21, 11 F, 10 M)	11.1 ± 1.2	13.5 ± 1.2
EL Group (n = 18, 10 F, 8 M)	11.2 ± 1.1	13.7 ± 1.3
UC2 Group (n = 15, 11 F, 4 M)	10.9 ± 1.1	13.0 ± 0.7

MA: Mandibular Advancement; EL: elastics; UC2: untreated Class II subjects; SD: standard deviation; F female; M male.

**Table 2:** Comparisons of descriptive statistics and baseline characteristics; ANOVA with Tukey’s post hoc test or rank-based ANOVA with Dunn’s post hoc test.

Variables	El Group (1) (n = 18)		MA Group (2) (n = 21)		Control Group (3) (n = 15)		*p*	Multiple Test Comparisons								
	Mean	SD	Mean	SD	Mean	SD		1 vs. 2			1 vs. 3			2 vs. 3		
Sagittal skeletal								Diff	*p*	95% Cl	Diff	*p*	95% Cl	Diff	*p*	95% Cl
SNA (°)	81.5	2.1	82.2	2.6	81.7	2.7	0.9	−0.66	0.75	−2.87 to 1.55	−0.16	0.99	−2.53 to 2.21	0.5	0.870	−1.9 to 2.9
SNB (°)	75.9	1.3	76.0	2.0	75.5	2.5	0.8	−0.09	0.98	−1.87 to 1.70	0.47	0.83	−1.45 to 2.38	0.5	0.836	−1.8 to 2.9
ANB (°)	6.2	1.3	5.4	1.4	6.0	1.4	0.4	0.82	0.24	−0.39 to 2.03	0.23	0.9	−1.07 to 1.52	−0.6	0.520	−1.9 to 0.7
WITS (mm)	2.8	1.2	2.7	1.8	2.7	2.7	0.9	0.09	0.00	−1.70 to 1.88	0.1	0.99	−0.81 to 2.01	0.0	1.000	−1.7 to 1.8
CoGn (mm)	103.3	6.7	101.9	6.5	98.7	5.7	0.2	1.36	0.82	−4.16 to 6.89	4.56	0.16	−1.34 to 10.48	3.2	0.257	−1.6 to 8.0
TVL-Pg’	−10.2	6.4	−8.9	4.6	−8.7	2.7	0.4	−1.21	0.75	−5.29 to 2.87	−1.49	0.69	−5.85 to 2.87	−0.2	0.977	−3.5 to 3.0
Vertical skeletal																
SN-Pal. Pl. (°)	7.6	1.7	7.4	2.6	7.2	3.7	0.14	1.2	0.27	−2.15 to 2.83	1.4	0.23	−0.6 to 3.22	0.2	0.970	−2.0 to 2.5
SN-Mand. Pl. (°)	33.5	4.4	32.0	4.0	32.1	4.7	0.4	2.50	0.38	−2.03 to 7.03	2.39	0.46	−2.47 to 7.24	−0.1	0.998	−4.2 to 4.0
Pal. Pl.-Mand. Pl. (°)	25.4	6.5	22.9	5.1	25.4	5.3	0.2	2.60	0.25	−1.3 to 6.5	0.1	0.1	−1.3 to 6.5	−2.5	0.436	−7.3 to 2.3
CoGoMe (°)	123.2	5.3	121.3	4.7	121.6	4.5	0.14	4.34	0.08	−0.38 to 9.06	4.03	0.14	−1.01 to 9.09	−0.3	0.985	−4.8 to 4.2
Dentoalveolar																
OVJ (mm)	4.9	1.6	5.2	2.1	6.1	2.3	0.5	−0.3	0.92	−2.10 to 1.5	−1.14	0.33	−3.08 to 0.79	−0.9	1.00	
OVB (mm)	2.2	1.7	4.7	1.6	2.9	1.1	<0.001	−2.49	<0.001	−3.82 to −1.15	−0.69	0.47	−2.13 to 0.74	1.8	0.003	0.5 to 3.0
U1 to Pal. Pl. (°)	111.8	5.1	112.0	9.6	112.2	6.1	0.9	−0.22	1	−6.95 to 6.51	−0.49	0.99	−7.69 to 6.71	−0.2	0.994	−6.7 to 6.2
L1 to Mand. Pl. (°)	101.2	10.1	100.2	9.0	97.1	7.3	0.3	0.94	0.95	−6.80 to 8.67	4.02	0.47	−4.26 to 12.30	3.1	0.420	−2.8 to 8.9

SD: standard deviation; Diff: difference; TVL: true vertical line; Pal. Pl.: palatal plane; Mand. Pl.: mandibular plane; 95% CI: 95% confidence interval; EL: elastics; MA: Mandibular Advancement.

**Table 3:** Descriptive statistics and statistical comparisons of the T2–T1 changes. Analysis of variance (ANOVA) with Tukey’s post hoc tests or ANOVA on ranks with Dunn’s post hoc tests.

Variables	El Group (1) (n = 18)		MA Group (2) (n = 21)		Control Group (3) (n = 15)		*p*	Multiple Test Comparisons								
	Mean	SD	Mean	SD	Mean	SD		1 vs. 2			1 vs. 3			2 vs. 3		
								Diff	*p*	95% Cl	Diff	*p*	95% Cl	Diff	*p*	95% Cl
ANB (°)	−2.2	1.7	−1.5	1.5	0.2	0.3	<0.001	−1.02	0.09	−2.16 to 0.13	−2.64	<0.01	−3.85 to −1.44	−1.7	0.001	−2.6 to −0.6
CoGn (mm)	5.5	2.0	8.3	3.1	3.3	1.2	<0.001	−2.8	<0.001	−4.82 to −1.71	2.2	0.002	0.82 to 4.14	5.0	0.000	4.30 to 7.2
TVL-Pg’	0.5	0.7	2	3.7	−1.6	3.3	0.13	−1.5	0.04	−5.35 to −0.27	2.1	0.13	−0.58 to 5.43	3.6	<0.001	2.33 to 7.59
Pal. Pl.^Mand.Pl. (°)	1.1	6.4	−0.5	3.1	0.6	0.8	0.004	3.24	0.04	0.09 to 6.39	0.98	0.75	−2.33 to 4.28	−2.6	0.000	−3.9 to −1.2
OVJ (mm)	−1.8	2.5	−1.5	7.0	0.0	0.0	0.003	0.42	0.84	−1.40 to 2.23	−2.25	0.02	−4.16 to −0.34	−2.6	0.000	−4.1 to −1.0
OVB (mm)	0.9	2.1	−1.0	7.9	0.7	1.6	<0.001	2.51	<0.001	1 to 4.02	0.56	0.67	−1.03 to 2.15	−1.9	0.001	−3.1 to −0.7
U1 to Pal. Pl. (°)	−3.2	9.2	−0.4	2.6	−0.7	1.5	0.3	−2.97	−8.84	2.90 to 0.44	−3.54	0.36	−9.71 to 2.64	−0.5		
L1 to Mand. Pl. (°)	1.6	3.3	1.4	3.2	0.4	1.1	0.2	−3.50	0.50	−11.05 to 4.04	−5.89	0.18	−13.83 to 2.05	−1.7	0.603	−6.0 to 2.6

SD: standard deviation; Diff: difference; TVL: true vertical line; Pal. Pl.: palatal plane; Mand. Pl.: mandibular plane; 95% CI: 95% confidence interval. EL: elastics; MA: Mandibular Advancement.

## Data Availability

The datasets used and/or analyzed during the current study are available from the corresponding author upon reasonable request.

## Abbreviations

The following abbreviations are used in this manuscript:

EL	elastics
MA	Mandibular Advancement
CVM	cervical vertebral maturation
T1	pre-treatment phase
T2	post-treatment phase
UC2	untreated Class II
IPR	interproximal reduction
ANOVA	analysis of variance
ICC	intraclass correlation coefficient
MME	method of moments’ estimator
